# Distinct MicroRNA Subcellular Size and Expression Patterns in Human Cancer Cells

**DOI:** 10.1155/2012/672462

**Published:** 2012-02-12

**Authors:** Beibei Chen, Bo Zhang, Huaxia Luo, Jiao Yuan, Geir Skogerbø, Runsheng Chen

**Affiliations:** ^1^Laboratory of Bioinformation and Noncoding RNA and Center for Systems Biology, Institute of Biophysics, Chinese Academy of Sciences, Beijing 100101, China; ^2^Graduate University of Chinese Academy of Sciences, Beijing 100080, China

## Abstract

*Introduction*. Small noncoding RNAs have important regulatory functions in different cell pathways. It is believed that most of them mainly play role in gene post-transcriptional regulation in the cytoplasm. Recent evidence suggests miRNA and siRNA activity in the nucleus. Here, we show distinct genome-wide sub-cellular localization distribution profiles of small noncoding RNAs in human breast cancer cells. *Methods*. We separated breast cancer cell nuclei from cytoplasm, and identified small RNA sequences using a high-throughput sequencing platform. To determine the relationship between miRNA sub-cellular distribution and cancer progression, we used microarray analysis to examine the miRNA expression levels in nucleus and cytoplasm of three human cell lines, one normal breast cell line and two breast cancer cell lines. Logistic regression and SVM were used for further analysis. *Results*. The sub-cellular distribution of small noncoding RNAs shows that numerous miRNAs and their isoforms (isomiR) not only locate to the cytoplasm but also appeare in the nucleus. Subsequent microarray analyses indicated that the miRNA nuclear-cytoplasmic-ratio is a significant characteristic of different cancer cell lines. *Conclusions*. Our results indicate that the sub-cellular distribution is important for miRNA function, and that the characterization of the small RNAs sub-cellular localizome may contribute to cancer research and diagnosis.

## 1. Introduction

Cancer is a syndrome with complex pathogeny and is one of the principal causes of mortality all over the world [1]. When a normal cell transforms to a tumor cell, a variety of genes change their expression characteristics. It is believed that the deregulation of oncogenes and tumor-suppressor-genes is one of the major causes of tumorigenesis. Present knowledge suggests that deregulation of gene expression consists of at least two aspects, abnormal gene expression levels and irregular localization of the gene products (i.e., mRNAs, proteins or noncoding RNAs). With the exception of a few reports concerned with the subcellular localization of the gene products of p53 and BRCA1 [2, 3], most focus has been on expression levels. Although recently an increasing number of noncoding RNA (ncRNA) (e.g., miR-21, miR-155, miR-122, and others) have been shown to act as oncogenes (oncomirs) or tumor-suppressor genes [4, 5], the sub-cellular localization of noncoding RNAs is not well known, and little effort have been directed at analyzing the sub-cellular localization regulation of microRNAs and other important noncoding RNAs [6].

Small noncoding RNAs, including microRNAs (miRNAs), short interfering RNAs (siRNAs), and Piwi-interacting RNAs (piRNAs), are important regulators of gene expression [7]. miRNAs and siRNAs guide sequence-specific cleavage, deadenylation, and translational repression of target mRNAs [8], whereas piRNAs appear to be specifically expressed in testes [9] and control retrotransposition activity in the mammalian germ line [10, 11]. MicroRNAs are cleaved from 70- to 100-nucleotide pre-miRNA hairpins, yielding 19- to 25-nucleotide long mature miRNAs [12]. One example of miRNA involvement in tumorigenesis is represented by miR-10b, which initiates tumour invasion and metastasis in breast cancer, one of the most common malignancies among women worldwide [13]. Early investigations suggested that mature microRNAs have a cytoplasmic localization in the cell. However, several lines of evidence point to the existence of active nuclear RNAi pathways in human cell nuclei. Recent research indicate that siRNA-induced transcriptional gene silencing through DNA methylation occur in various human cell types [14, 15], and small nuclear RNAs (e.g., 7SK and U6) have been downregulated by ectopic siRNA activity [16]. The presence of the RNA-induced silencing complex (RISC) component AGO2 in the nucleus has been demonstrated [17], and at least one microRNA (miR-29b) is present in the nucleus [18]. However, the mechanisms by which miRNAs and RISCs are programmed to localize in and function in the nucleus are not known in detail [19].

In order to investigate the sub-cellular distribution of small noncoding RNAs and its relation to cancer, three different cell lines were used: normal breast cells (MCF-10A), noninvasive breast cancer cells (MCF-7), and invasive breast cancer cells (MDA-MB-231). Small RNA profiles from nuclei and cytoplasm of MDA-MB-231 were obtained using the Illumina high-throughput sequencing platform (Solexa) [20]. Based on the small RNA sequences detected in MDA-MB-231, a RNA microarray was designed to obtain a sub-cellular localization profile of the small RNAs in the three cell lines, and miRNA “nuclear-cytoplasmic ratios” (NCRs) were calculated for all three cell lines to explore whether the changes in sub-cellular localization relate to cancer progression.

## 2. Materials and Methods

### 2.1. Small RNA Library Preparation and High-Throughput Sequencing

The human breast cancer cell lines MCF-7, MDA-MB-231 and the human mammary epithelial cell line MCF-10A were obtained from the American Type Culture Collection (ATCC, Rockville, MD, USA). MCF-10A cells were cultured in DMEM-F12 (Life Technologies) supplemented with 5% horse serum, 0.5 *μ*g/mL hydrocortisone (Sigma), 10 *μ*g/mL insulin (Sigma), 20 ng/mL epidermal growth factor (Sigma), 100 *μ*g/mL penicillin and 100 *μ*g/mL streptomycin. Other cells were grown in DMEM (Life Technologies) supplemented with 100 *μ*g/mL penicillin, 100 *μ*g/mL streptomycin, and 10% heat-inactivated FBS at 37°C in a humidified atmosphere containing 5% CO_2_. Transfection of tumor cells with miRNAs (3.5 × 10^6^) was performed with lipofectamine 2000 (Invitrogen) according to the manufacturer's instructions.

Sub-cellular fractioning was executed according to the sub-cellular fractionation protocol provided by Abcam [21]. Total RNA was extracted from the sub-cellular fractions with Trizol (Invitrogen). Immediately following RNA precipitation, construction of small RNA cDNA libraries for SOLEXA high-throughput sequencing was initiated from 20 *μ*g each of the nuclear and cytoplasmic small RNA isolates. The RNA was size-fractioned using 15% TBU PAGE, and a gel fragment corresponding to RNAs of 17 to 30 nucleotides was excised, and the RNA eluted. The eluted RNAs were precipitated with ethanol and resuspended in diethyl pyrocarbonate-treated deionized water. The gel-purified small RNAs were ligated sequentially first to 5′ end and then to 3′ end RNA oligonucleotide adapters using T4 RNA ligase (10 units/*μ*L) (Promega, Madison, WI). The 5′ end RNA adapter (5′GUUCAGAGUUCUACAGUCCGACGAUC3′) possessed 5′ and 3′ hydroxyl groups, whereas the 3′ RNA adapter (5′-pUCGUAUGCCGUCUUCUGCUUGidT-3′) possessed a 5′ monophosphate and a 3′ inverted deoxythymidine (idT). The small RNAs were first legated to the 5′ RNA adapter, and the ligation products were gel-eluted and ligated to the 3′ RNA adapter. The final ligation products were then used as templates in a reverse transcription (RT) reaction using the RT-primer (5′CAAGCAGAAGACGGCATACGA3′) and Superscript II reverse transcriptase (Invitrogen, Carlsbad, CA, USA) according to the instructions of the Illumina manufacturer. This was followed by a 15 cycle PCR amplification step using the PCR reverse (5′-AATGATACGGCGACCACCGACAGGTTCAGAGTTCTACAGTCCGA-3′) and forward (5′-CAAGCAGAAGACGGCATACGA-3′) primers and Phusion hot-start high-fidelity DNA polymerase (New England Biolabs, Cambridge, MA, USA). All oligonucleotides were provided by Illumina (San Diego, CA, USA). Lastly, the amplification products were separated by electrophoresis on a 6% polyacrylamide gel in TBE buffer and eluted, precipitated using ethanol, and resuspended in nuclease-free water. The purified PCR products were quantified on the Agilent DNA 1000 chip and diluted for sequencing on the Illumina 1G.

### 2.2. Small RNA Genomic Mapping and Annotation

All the sequences were aligned to the human reference genome hg17 (retrieved from the University of California, Santa Cruz (UCSC)) web site [22] using Mega BLAST (version 2.2.9), and only the ones of which more than 90% of the nucleotides mapped to the genome sequences were retained. For each read, the longest alignment was determined, and the shorter reads that mapped to the same position were stored as an isoforms group. The mapped small RNA sequences were used for further analysis if the corresponding transcript was detected at least three times. The information used for annotation derived from RefSeq genes, RepeatMasker, and sno tables from the UCSC web site. Sequences of all known miRNA genes were downloaded from miRBase release 12.0, and other known ncRNAs were downloaded from the most relevant databases [23–26].

A BLAST (blastn program) search was then performed on the downloaded material using our small RNA sequences as queries, and hits were defined with a 90% match criterion (including indels). Sequences overlapping annotations from the downloaded databases were classified into the following classes in the listed order: miRNA, piRNA, tRNA, rRNA, snoRNA, snRNA, other known noncoding RNA, genomic repeats, and known protein-coding gene (exon or intron). Mapped sequences that did not overlap any of these annotations were automatically classified as “unknown”.

### 2.3. Novel MicroRNAs Prediction

Candidate miRNA gene sequences were identified from uniquely mapped small RNA sequences (reads ≥ 3) which lack annotations in above databases. Then 240 bp of genomic sequence flanking each side of these candidate sites was extracted. A previously published method (“MiRscan” [27]) was used to score the candidate pre-miRNA sequences and identify the most likely pre-miRNA hairpins and candidate microRNAs.

MiRscan was trained with the known human microRNAs and pre-miRNA sequences in miRBase (release 12.0) to gain miRNA score matrix. According to this score matrix, each candidate pre-miRNA was assigned a score to indicate similarity to hairpins of experimentally verified human microRNAs. With a cutoff score of 7.200 (*P*-value < 0.005), 48 novel microRNAs were obtained from the nucleus and 31 from the cytoplasm.

### 2.4. Microarray Assay and Nuclear-Cytoplasmic Ratio Calculation

The *μ*Paraflo microRNA microarray assay was used to determine the miRNA nuclear-cytoplasmic ratio (NCR) in MDA-MB-231, MCF-7, and MCF-10a cells. Nuclear and cytoplasmic RNA was extracted as described previously. Probes were designed corresponding to more than one thousand of the detected small RNAs in MDA-MB-231, and nuclear and cytoplasmic small RNA samples were labeled with Cy5 and Cy3, respectively, thus enabling direct calculation NCR from the signal intensity ratios (for further details see Supplemental Methods available online at doi:10.1155/2012/672462). All data in this work are MIAME compliant.

### 2.5. MicroRNA Classification Based on NCR

To distinguish the microRNAs by their expression and localization profile variation, we defined a microRNA integrative variation trend between two cell lines (*V*_*θ*_).  Figure 5 shows an example of *V*_*θ*_ for MCF-10A and MCF-7 (denoted as *V*_*θ*_*c*_), calculated as

(1) $$V_{\theta \_c}=\tan\left(\theta_{c}\right)=\left[\frac{\Delta_{N}}{\Delta_{C}}\right]=\left(\frac{N_{\text{MCF}10\text{A}}-N_{\text{MCF}7}}{C_{\text{MCF}10\text{A}}-C_{\text{MCF}7}}\right),$$

where the Δ_*N*_ = (*N*_MCF-10A_ − *N*_MCF-7_) is the variation in the nuclear expression intensity of a given microRNA, and the Δ_*C*_ = (*C*_MCF-10A_ − *C*_MCF-7_) is the variation in the cytoplasmic expression intensity of the same microRNA. The ratio of nuclear to cytoplasmic expression variation thus represents the microRNA integrative variation trend. For each microRNA, three variation trends, *V*_*θ*_*a*_, *V*_*θ*_*b*_, and *V*_*θ*_*c*_ were calculated, representing the variation trends between MCF-7 and MDA-MB-231, MDA-MB-231 and MCF-10A, MCF-10A, and MCF-7, respectively. For all microRNAs that appeared in all the three cell lines, we calculated the Pearson correlation coefficient for microRNA pairs based on the vectors (*V*_*θ*_*a*_, *V*_*θ*_*b*_, and *V*_*θ*_*c*_). The tri-NCR groups were normalized by setting the highest intensity to 100. MicroRNAs with similar NCR positions in each cell line were grouped together (e.g., miR-16 and miR-20a, miR-93 and miR106b; Figure 4).

In Figure 5, “*c*” is the variation track from MCF-10A to MCF-7 of the miR-21. Thus, we defined the integrative variation trends for miR-21 from MCF-10A to MCF-7 as

(2) $$V_{\theta \_c}=\tan\left(\theta_{c}\right)=\left[\frac{\Delta_{N}}{\Delta_{C}}\right]=\left(\frac{N_{\text{MCF}7}-N_{\text{MCF}10\text{A}}}{C_{\text{MCF}7}-C_{\text{MCF}10\text{A}}}\right).$$

For each microRNAs, in each cell state, (*V*_*θ*_) has at least two characteristics, the expression intensity and the sub-cellular localization. Here, we define the microRNA expression intensity in the cell as

(3) $$r=\sqrt{N_{\text{intensity}}^{2}+C_{\text{intensity}}^{2}},$$

and the microRNA sub-cellular localization (NCR) profile parameter in the cell as

(4) $$\text{theta}=\arctan\left(\frac{N_{\text{intensity}}}{C_{\text{intensity}}}\right),$$

where *N*_intensity_ and *C*_intensity_ are the nuclear and cytoplasmic expression intensities, respectively.

### 2.6. SVM Classification of Different Breast Cancer Cell Lines

In a further analysis, we explored the connection between the cancer development states and the microRNA NCRs. In order to simultaneously utilise both the expression intensity and the NCR of the microRNAs in each cell line, we transferred the rectangular coordinate to angular coordinates (Figure 5 shows an example for *r*_MCF-7_ and *θ*_MFC7_).  Figure 3(a) demonstrates the angular coordinate of a given miRNA. A binary classification SVM (ksvm in the *R* package kernel lab) was used to further classify them. Using the built-in linear kernel function in the package which maps the vector to a higher dimensional space, a hyperplane can be found so that the distance from it to the nearest data point on each side (microRNAs from MDA-MB-231 and MCF-7) is maximized. At the same time, the contour lines of the values generated by the kernel function are nearly vertical, suggesting that the decisive variable of the kernel is NCR, and that the NCR value can be used to distinguish the breast cancer cell lines (Figure 3(b)). 

### 2.7. Statistic Analysis of the MicroRNA Isoform Size Distribution

To test if sequence length is significantly correlated with miRNA sub-cellular localization, a Boolean variable (1 and 0 indicating that a sequence is located in the nucleus and cytoplasm, resp.) was used as the response variable and the length of the sequences in each dataset as the predictor. The logistic regression showed the significance level of the coefficient estimate for the predictor (*P* < 2*e* ^−16^). For one nucleotide increase in sequence length, the log odds of the sequence staying in nucleus increased by 0.124. A post-chi-square test of the difference between the current model and the null model also show the logistic model fitted well (*P* < 2.12*e* ^−16^). These results suggested that the size of mature microRNAs and their isoforms could be correlated with the sub-cellular localization of the mature microRNAs.

## 3. Results

### 3.1. Genomewide Detection and Annotation of Nuclear and Cytoplasmic Small RNAs in Human Breast Cancer Cells

**Figure 1:**
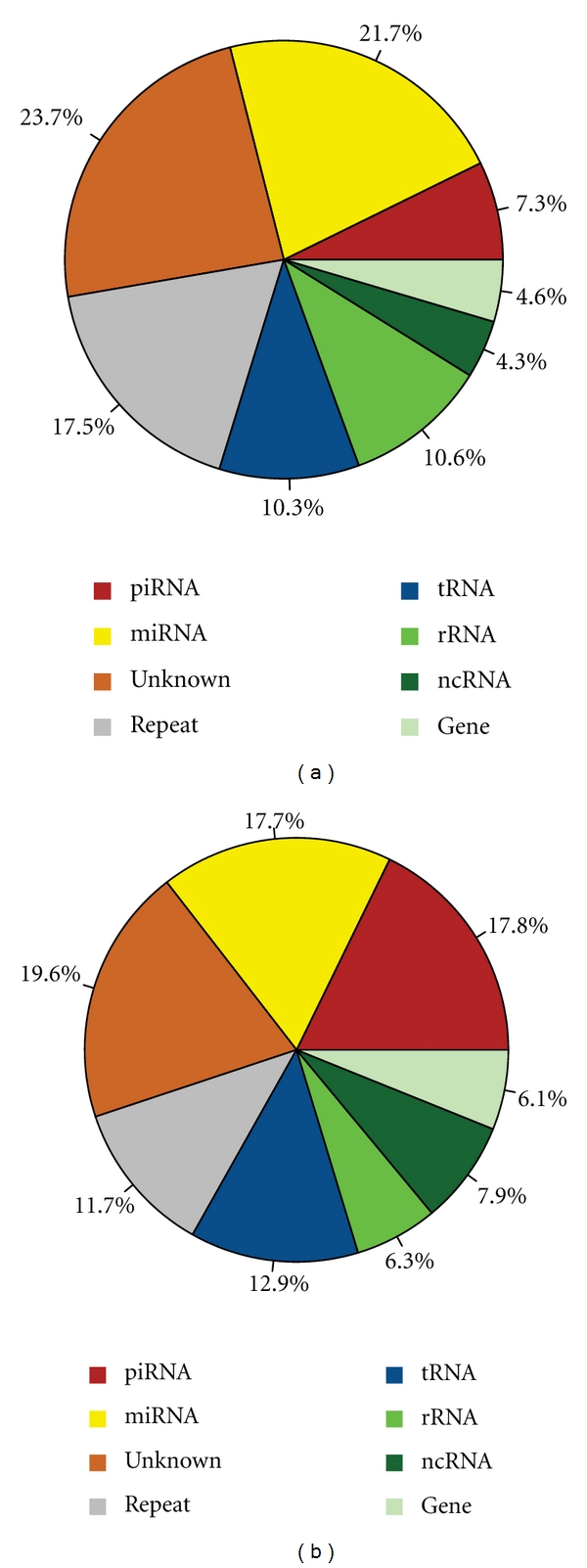
Distribution of small RNA sequences. (a) Distribution of 7498 cytoplasmic small RNA sequences. (b) Distribution of 15255 nuclear small RNA sequences. Almost one-fifth of nuclear small RNAs was annotated as piRNAs.

High-throughput sequencing of small RNA samples in MDA-MB-231 human breast cancer cells produced 3,053,680 and 3,216,761 unfiltered small RNA reads from nuclear and cytoplasmic RNA isolates, respectively (Supplemental Figure  S1). After filtering and mapping (Supplemental Table  S1), the nuclear and cytoplasmic samples yielded 15,255 and 7,498 reliable small RNA sequences, respectively (Figure 1). Among these, we found 5,260 and 2,695 small RNAs that could be mapped to unique genomic positions (Supplemental Table  S2). The 5,260 nuclear sequences could be divided into three roughly equal fractions consisting of 1,646 sequences (31%) annotated as small ncRNAs, 1,808 sequences (34%) corresponding to tRNAs, rRNAs, other ncRNAs and mRNA fragments, and 1,806 sequences (34%) corresponding to nonannotated sequences. The 1,806 non-annotated sequences may represent novel miRNAs, piRNAs, siRNAs, or possibly new types of small RNAs [28]. More than 60% of the uniquely mapped small RNAs loci are intergenic and about 15% originated from coding gene introns (Supplemental Table S3 and Figure  S2). Close to 3% (171 sequences) of the nuclear uniquely mapped sequences are annotated as piRNAs, which previously have only been reported to be expressed in mammalian germline cells [11]. Using all mapped sequences from the nuclear sample (15,255) as queries in a BLAST search against all known piRNAs, we found that 2,871 of the sequences can be mapped to 118 known piRNAs (Supplemental Table  S4).

To distinguish putative novel miRNAs and endo-siRNAs [29, 30], which are generally 18–25 nucleotides long, from possible piRNAs which are slightly longer (26–30 nt), we divided the RNAs into a short and a long fraction. Analysis of the short fraction loci with in-house and public algorithms (see Section 2) identified 48 putative novel microRNA genes that are expressed in the MDA-MB-231 cell line (Supplemental Table  S5). Recent results from high-throughput sequencing have demonstrated that experimentally obtained miRNAs sequences frequently deviate from their corresponding reference sequences in miRBase, suggesting the existence of multiple mature miRNA variants (isomiRs) [20]. Similar variation at the 5′ and 3′ ends of known miRNAs was also observed in our study (Supplemental Table  S6). Although the possible functions of miRNA isoforms are unknown, it is evident that 5′ end variation in a miRNA could alter the active seed sequence and lead to differences in target specificity [31].

### 3.2. MicroRNA and Isoform Size Correlates with Subcellular Localization

**Figure 2:**
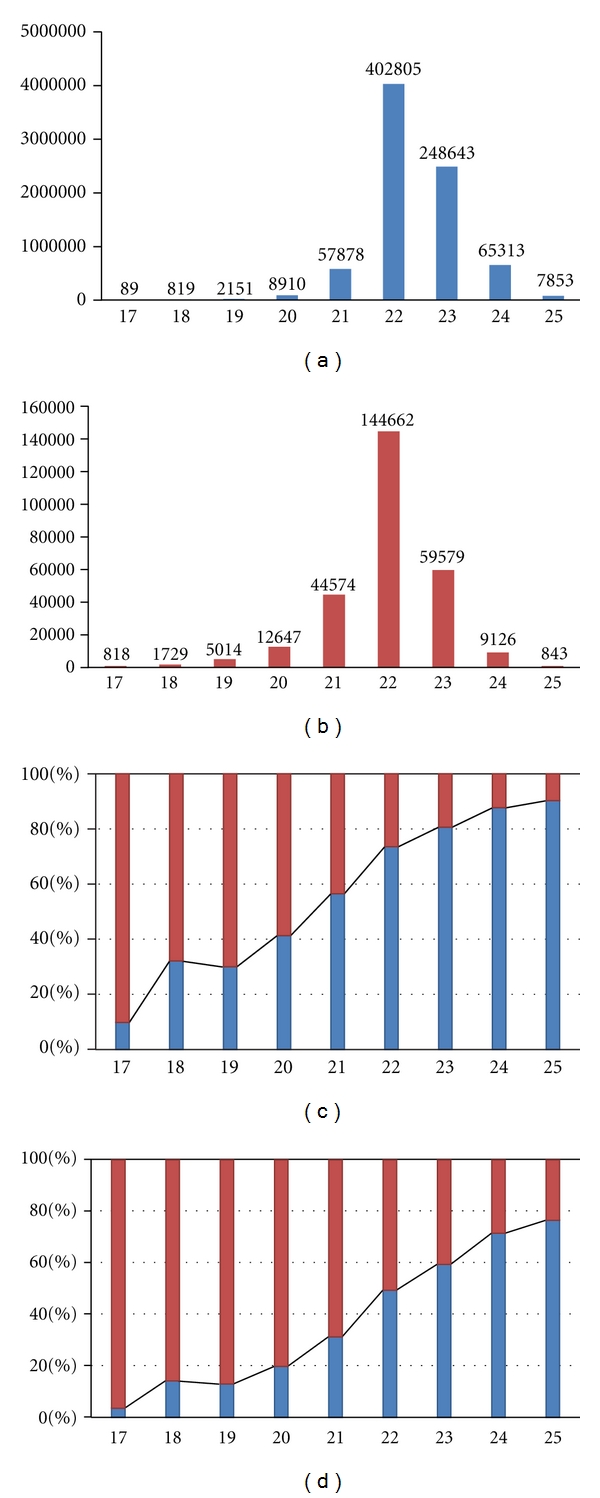
MicroRNA size correlates with sub-cellular distribution. (a) Size distribution of nuclear microRNA reads. (b) Size distribution of cytoplasmic microRNA reads. (c) Proportion cytoplasmic (red) and nuclear (blue) of raw reads of different sizes (17 to 25 nt). (d) Proportion cytoplasmic (red) and nuclear (blue) of reads of different sizes (17 to 25 nt) number proportion of every size (17 to 25 nt) for cytoplasmic (Red) to nuclear (Blue). The proportion of nuclear reads increases with the increasing size of microRNAs from 17 to 25 nt.

MicroRNAs and RISCs have been found in both cytoplasm and nucleus, but the mechanisms specifying their sub-cellular localization and transport are not clear. A hexanucleotide element directing miRNA nuclear import appears only to be relevant for miR-29b [18]. To clarify what possibly determines the microRNA sub-cellular localization profile, we compared small RNA sequences annotated as known microRNAs, obtained from nuclear and cytoplasmic extracts of MDA-MB-231 cells. No specific sequence motif was found that could explain the difference in sub-cellular localization, but the sequence size distributions of the two sub-cellular miRNAs population differed clearly. To further quantify this difference, the lengths of all the isoforms of every known microRNA in the nuclear and cytoplasmic data sets were collected, and a logistic regression showed a significant (*P* < 2.2 × 10^−16^) difference in length distribution of miRNAs and isomirs found in the nuclear and cytoplasmic compartments (see Section 2; Figure 2).

### 3.3. MicroRNAs Show Varying Sub-Cellular Expression Profiles in Human Breast Cancer Cell Lines

**Figure 3:**
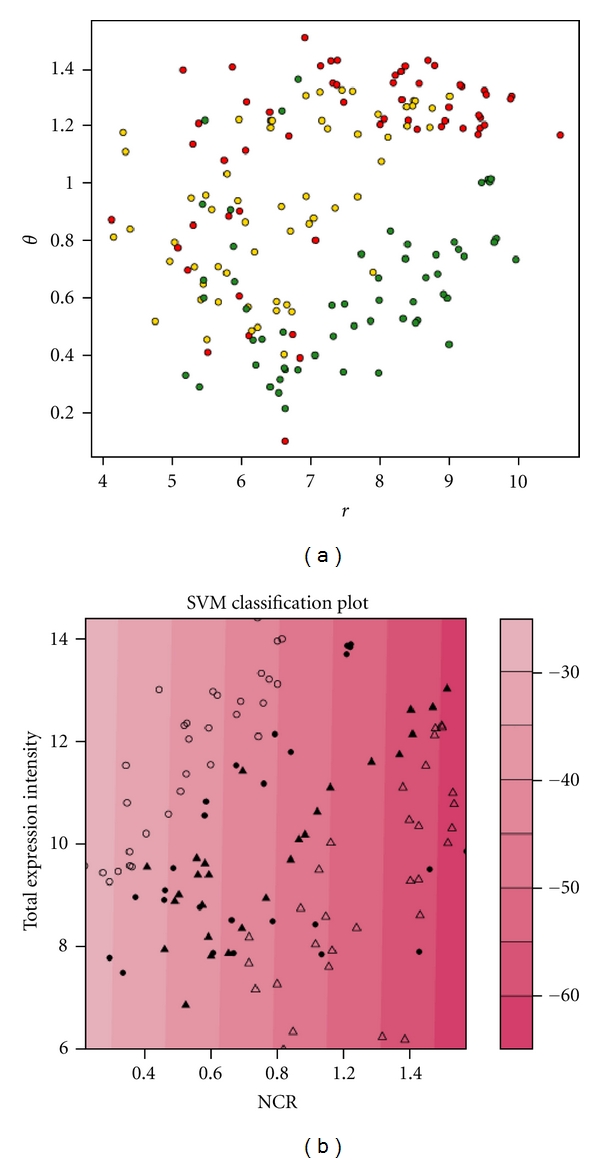
MicroRNA integrated expression and location characteristics in three breast (cancer) cells lines. The figure only includes microRNAs with normalized cytoplasmic and nuclear signal intensities above 50. (a) MicroRNAs sorted by their total expression (*r*) and NCR (nuclear-cytoplasmic ratio; theta). (For definition of “*r*” and “*θ*”, see Figure 5). The red, green, and yellow dots represent the MDA-MB-231, MCF-7, and MCF-10A cell lines, respectively. (b) SVM classification of the MDA-MB-231 (circles) and MCF-7 (triangles) breast cancer cell lines, based on the microRNA total expression (signal intensity) and NCR (nuclear-cytoplasmic ratio). The scale bar on the right shows the value generated by the kernel function of the corresponding vectors. This figure suggests that the microRNA NCR is the decisive variable distinguishing the two cancer cell lines.

Translocation between nucleus and cytoplasm appears to be a functional aspect of certain oncogenes and tumour suppressors, such as p53 and BRCA1 [2, 3]. In order to investigate nuclear and cytoplasmic distributions of small RNAs in normal breast cells (MCF-10A), noninvasive breast cancer cells (MCF-7) and invasive breast cancer cells (MDA-MB-231), we designed a small RNA microarray based on the small RNA sequences detected in MDA-MB-231 to obtain a localization profile of the small RNAs. We calculated their “nuclear-cytoplasmicratio” (NCR) and found that the NCR values of a large number of miRNAs varied considerably between different cell lines (Supplemental Table  S7). It suggested that the miRNA sub-cellular localization may be correlated with the breast cancer progression. In some cases, the same microRNA presented similar overall expression levels in the three breast cell lines but showed distinct differences in their sub-cellular expression concentrations. Taken together, these data suggested that an integrative approach considering both the overall and sub-cellular expression profiles of miRNAs might be valuable in distinguishing different tumor cell lines (Figure 3).

## 4. Discussion

MicroRNA expression profiles are gaining importance as a means of accurately distinguishing different types of cancers [1, 32]. When combined with target prediction, they can also be used to identify miRNA regulated pathways [33], and in some cases differential miRNAs expression has been shown to influence the suppression of certain types of cancer [34]. Recently, evidence indicates that miRNAs not only are present in the cytoplasm but also in the nucleus. [35, 36]. Our findings provide new clues for identifying functions of cancer-related noncoding RNA genes. The spatial distribution of miRNAs, as indicated by their NCR patterns, may serve as future markers of cancer and malignancy states.

Meanwhile, based on our observation that the small RNA size distributions are different in the nuclear and cytoplasmic cellular compartments, and the present hypothesis that the Argonaute protein or the RISC is involved in shuttling small RNAs between the nucleus and cytoplasm, we would like to suggest the following hypothesis that loading of longer miRNAs or siRNAs onto AGO2 might induce structural changes in the RISC or otherwise provide the signal to specify the import of the RNA-protein complex into the nucleus. In the nucleus, miRNAs or siRNAs might act as guiding molecules in various functional pathways such as alternative splicing and epigenetic sites recognizing [37]. Moreover, since miRNAs in the cytoplasm generally target sites in the 3′ UTR of mRNAs, and 3′ UTR cis-elements have been reported to control the sub-cellular localization of mRNAs [38], the aberrant localization of miRNA may subsequently result in abnormal sub-cellular localization of mRNAs or proteins.

In animal testis cells, piRNAs have been shown to lead epigenetic modification to chromatin, including DNA methylation and histone modification [39]. However, the biological functions of short small RNAs (<25 nt) in the nucleus are still unclear. The fact that a number of miRNAs showed different sub-cellular distributions in different cell lines implies that “traditional” miRNA expression profiles may not be a complete reflection of the actual miRNA expression patterns. Furthermore, while cytoplasmic miRNAs may regulate gene expression at the posttranscription level by binding to 3′ UTR of mRNAs, current target predication methods based on 3′ UTR-seed matching may not be relevant to miRNAs that predominantly appear in the nucleus.

**Figure 4:**
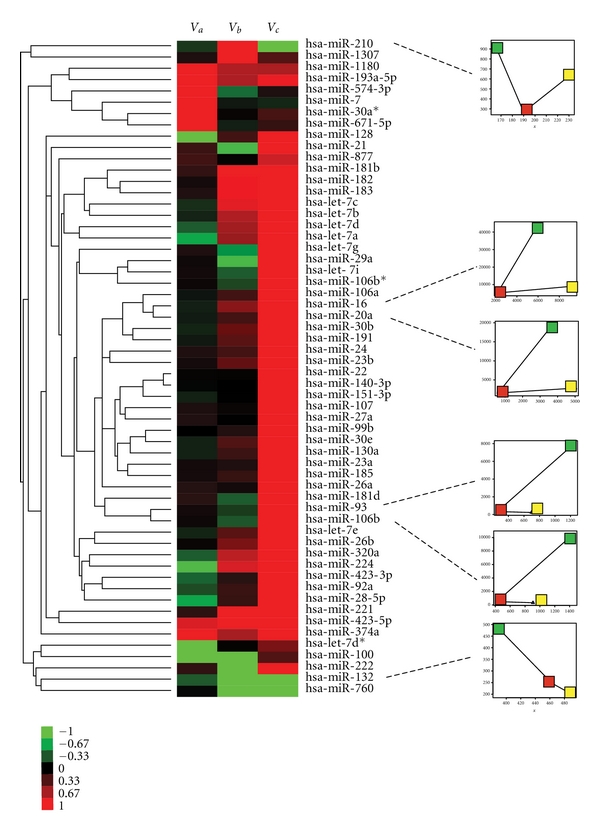
Clustering of the microRNAs by nuclecytoplasmic ratios (NCR) in three cell lines. Hierarchical clustering of microRNAs based on their nucleocytoplasmic integrative variation tendency (See Section 2), and the heatmap of miRNA NCR variation from three cell lines are shown. The small graphs on the right represent cytoplasmic (*x*-axis) and nuclear (*y*-axis) expression intensity of six selected miRNAs The red, yellow, and green dots are the MDA-MB-231, MCF-7, and MCF-10A cell lines, respectively.

**Figure 5:**
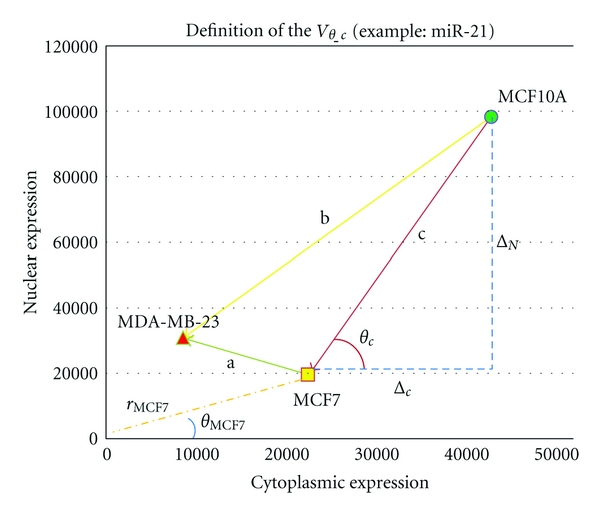
Definition of the microRNA integrative variation trend (*V*_*θ*_).

The NCR values of many miRNAs varied among three cell lines, as well as the expression of a number of miRNAs (Table 1). The expression level of miR-27b in the nucleus of MDA-MB231 cell line was almost 2 fold higher than in MCF-7 cell line; however, the cytoplasmic miR-27b levels were similar in the two cell lines. Moreover, the higher miR-27b expression in nucleus of MDA-MB231 cell line was in accordance with the result present above. Similarly, the nuclear expression of miR-29c was also significantly higher (*∼*17.8 fold) in MDA-MB231 than in MCF-7, compared to only about 4.7 fold difference in cytoplasmic expression. In contrast, the sub-cellular distribution of miR-29a was opposite to that of miR-29c in these two cell lines. In the MDA-MB231 cell line, the nuclear expression of miR-29a was almost 12.6 fold higher than in MCF-7, while the MDA-MB231 cytoplasmic expression miR-29a level was less than half that of MCF-7. These results suggest that in addition to altered protein sub-cellular distributions, the sub-cellular localization of regulative RNA molecules may also be important in cancer. Although the mechanism by which these nuclear small RNAs affect cancer is poorly understood, the sub-cellular miRNA distributions may be a future tool in cancer cell research and diagnosis (Figure 4). 

## 5. Conclusions

Using high-throughput sequencing and microarray techniques, we have identified 79 new miRNAs candidates and revealed distinct miRNA sub-cellular localization patterns in three different breast cancer cell lines. Moreover, the length distribution of miRNA isoforms varies in the different sub-cellular fraction, longer and shorter isoforms tending towards nuclear and cytoplasmic localizations, respectively. These facts suggest that the miRNA sub-cellular localization may play an important role in cancer diagnosis and research. 

## Supplementary Material

Figure S1. Length distribution of small RNAs in MDA-MB-231.
Figure S2. The small RNA genome location distribution near or within the coding genes.
Figure S3. Western bolt. Analysis of expression of Lamin A/C, GAPDH and beta-action in the extracts of nucleus, cytoplasma and whole cell.
Table S1. Total sequencing small RNAs genome mapping.
Table S2. Small RNA sequences annotation.
Table S3. The relationship between small RNAs loci and coding genes loci.
Table S4. The nuclear small RNAs annotated as known piRNAs.
Table S5. Predicted new microRNAs.
Table S6. Isoforms of microRNAs.
Table S7. MicroRNAs NCR values varied significantly between different cell lines.

## Figures and Tables

**Table 1:** Expression level and sub-cellular distributions of miRNA in two breast cancer cell lines.

miRNA	MDA-MB231				MCF-7			
	Total Reads*	NCR (log2)	*N* reads**	*C* reads**	Total Reads*	NCR (log2)	*N* reads**	*C* reads**
Hsa-miR-27b	56565	1.73	43463	13102	24084	−1.09	7700	16384
Hsa-miR-29a	5811	3.05	5185	626	1976	−1.93	410	1565
Hsa-miR-29c	172226	~0	86113	86113	23259	−1.93	4835	18424

The sequencing data for MDA-MB231 and MCF-7 in GSE16579 were downloaded from the Gene Expression Omnibus (GEO). Adapter sequences were removed from reads. Filtered reads with lengths from 18 nt to 25 nt were annotated using MirBase, v.16.0 by BLAST with at 100% identify (0 mismatch).

*Total Reads obtained from GSE16579.

**N- and *C*-reads are reads number in nucleus and cytoplasm, respectively. These were calculated from Total Reads and the NCR.

## Abbreviation

NCR: Nuclear-cytoplasmic ratio.
